# High-Speed Hardware Architecture Based on Error Detection for KECCAK

**DOI:** 10.3390/mi14061129

**Published:** 2023-05-27

**Authors:** Hassen Mestiri, Imen Barraj

**Affiliations:** 1 Department of Computer Engineering, College of Computer Engineering and Sciences, Prince Sattam bin Abdulaziz University, Al-Kharj 11942, Saudi Arabia; 2 Higher Institute of Applied Sciences and Technology of Sousse, University of Sousse, Sousse 4002, Tunisia; 3 Electronics and Micro-Electronics Laboratory, Faculty of Sciences of Monastir, University of Monastir, Monastir 5000, Tunisia; 4 Systems Integration & Emerging Energies (SI2E), Electrical Engineering Department, National Engineers School of Sfax, University of Sfax, Sfax 3029, Tunisia; 5 Higher Institute of Computer Science and Multimedia of Gabes (ISIMG), University of Gabes, Gabes 6029, Tunisia

**Keywords:** cryptographic circuits, security, KECCAK, fault attacks, fault detection

## Abstract

The hash function KECCAK integrity algorithm is implemented in cryptographic systems to provide high security for any circuit requiring integrity and protect the transmitted data. Fault attacks, which can extricate confidential data, are one of the most effective physical attacks against KECCAK hardware. Several KECCAK fault detection systems have been proposed to counteract fault attacks. The present research proposes a modified KECCAK architecture and scrambling algorithm to protect against fault injection attacks. Thus, the KECCAK round is modified so that it consists of two parts with input and pipeline registers. The scheme is independent of the KECCAK design. Iterative and pipeline designs are both protected by it. To test the resilience of the suggested detection system approach fault attacks, we conduct permanent as well as transient fault attacks, and we evaluate the fault detection capabilities (99.9999% for transient faults and 99.999905% for permanent faults). The KECCAK fault detection scheme is modeled using VHDL language and implemented on an FPGA hardware board. The experimental results show that our technique effectively secures the KECCAK design. It can be carried out with little difficulty. In addition, the experimental FPGA results demonstrate the proposed KECCAK detection scheme’s low area burden, high efficiency and working frequency.

## 1. Introduction

In the current world of online banking, online shopping, e-mail, and other sensitive digital interactions, cryptography has become an indispensable instrument for protecting the confidentiality of data transmissions. Hash functions form the basis of a large number of widely used cryptographic techniques, including Transport Layer Security (TLS), the Digital Signature Standard (DSS), encryption algorithms, numerous random number generation algorithms, Internet Protocol Security (IPSec) protocols, all or nothing transforms, and password storage mechanisms. The well-known SHA-1 algorithm has been significantly degraded. After the widely used SHA-1 hash algorithm became less secure and there were worries about the SHA-2 family of algorithms with a similar structure, the US National Institute of Standards and Technology (NIST) held a public competition to find a new cryptographic hash function standard to replace SHA-2. The KECCAK hash function was submitted to the SHA-3 competition and is one of the five remaining candidate functions. After three rounds of evaluation, KECCAK was selected as the winning algorithm for this standard in 2012. In October 2012, the competition concluded with the KECCAK algorithm as the victor. By August 2015, the final version of the new SHA-3 standard was published [1].

The KECCAK algorithm is utilized in daily systems to protect data privacy and assure the integrity of data exchange. Enhancing the performance of a KECCAK circuit implemented in embedded circuits is a significant challenge. Currently, the KECCAK algorithm is used in a wide range of applications requiring high security, such as smart cards and mobile communication [2,3,4,5,6,7,8,9]. Consequently, there is a need to enhance the KECCAK algorithm’s resilience against multiple physical attacks, such as fault attacks.

Fault attacks extract private information by inducing faults into the KECCAK architecture. Malicious and spontaneous fault injections reduce the KECCAK’s resilience and may result in leakage of secure data in unprotected implementations. The introduction of malicious faults is caused by voltage/clock glitches and electromagnetic radiation, as well as the surrounding environment. The KECCAK implementation on hardware is sensitive to these errors. More importantly, the introduced errors result in an incorrect KECCAK state, rendering the integrity message output untrustworthy. So far, only a handful of fault detection algorithms have been developed to increase the KECCAK implementation’s resilience [10,11,12,13,14,15,16,17,18,19].

Mestiri et al. proposed in [10] an effective error detection technique based on modifying the KECCAK architecture. The proposed system is not tied specifically to the KECCAK method of implementation. This means that pipeline and iterative designs may both benefit from it. The authors perform experimental attacks on their implementation to analyze it against fault injection attacks, and they estimate the fault detection capabilities to be about 99.997%. The KECCAK detection approach was developed using the VHDL hardware language, and the FPGA results demonstrate that their system can successfully defend KECCAK against fault attacks when implemented in hardware.

In order to offer a high degree of protection against fault attacks, an efficient error detection approach based on the bytes’ permutation technique has been developed in [11]. To investigate the robustness of the proposed detection technique against fault attacks, the authors conducted fault injection simulations and demonstrated that the fault coverage is around 99.996%. They have outlined the suggested detection technique, and the study of FPGA indicates that the scheme may be simply implemented with minimal complexity and effectively defend KECCAK against fault attacks.

Luo et al. present in [12] a technique for error detection in KECCAK based on parity testing that is both straightforward and effective. In addition, they offer optimized designs as a means of further enhancing the effectiveness of the proposed approach. In order to determine whether or not the suggested system has fault coverage, they first design it in VHDL and then simulate fault injection at the gate level. The results indicate that our approach provides high fault coverage for hardware implementations while placing only a very little burden on the resources available.

In this paper, we conduct a comprehensive fault analysis to evaluate the impact of fault attacks on the KECCAK implementation so that we can ensure a high level of protection against them. Moreover, we provide a reliable error detection system that improves upon the original KECCAK design. We summarize our contributions as:

- The KECCAK architecture is going to be modified according to the plan. Each KECCAK round is broken up into two separate half rounds. Therefore, the first half of the round is checked for errors at the same point in time during the clock cycle that the second part of the round is being carried out, and vice versa.
- In order to ensure the safety of all KECCAK operations, we suggest an updated architecture for KECCAK, which will result in a new fault detection method. In addition, we apply the scrambling technique to increase the degree of security provided by KECCAK. It is essential to point out that in comparison to its equivalents, our approach has a greater clock frequency, reduced area hardware requirements, and less throughput degradation.
- We provide proof that the fault detection technique that was suggested for the KECCAK identifies all naturally occurring as well as maliciously introduced faults. For this reason, we undertake fault attacks on our suggested architecture in every feasible location, including the error comparator detection and the integrity structure. This allows us to accomplish our goal. Using our simulation attacks, we demonstrate that the proposed scheme achieves 99.999905%, 99.9999% and 100% fault coverage for permanent faults, transient multiple and single fault, respectively. This is accomplished after injecting 2,000,000 multiple random and burst, single and permanent faults.
- Finally, we build both the unprotected and the proposed protected KECCAK architecture on FPGA, and we extract and compare the frequency and area overheads, as well as the throughput and efficiency deterioration caused by each. In comparison to the most current KECCAK error detection systems, the FPGA hardware implementation results reveal that our design has reduced throughput, area overhead and efficiency degradation, as well as a higher frequency.

The remainder of the paper is structured as follows: The second section provides background information. Section 3 presents the KECCAK implementation. In Section 4, we present the KECCAK fault analysis results. The fifth section describes the architecture of the proposed scheme for KECCAK. Section 6 compares and discusses the fault coverages. The results and comparisons of hardware implementation are presented in Section 7. The paper concludes with Section 8.

## 2. Background

### 2.1. KECCAK Algorithm

The Keccak algorithm relies heavily on the permutation f as its foundation. This function is often used to KECCAK states that have a constant length of b = c + r bits (r: bit-rate, c: capacity). The KECCAK data speed rose in direct proportion to the bit-rate r, but the KECCAK security level increased in direct proportion to the capacity c. An initial padding operation is performed on the KECCAK input message in order to generate a new input message with a length that is a multiple of r. Following this, there are five stages that make up each KECCAK round when it comes to the assimilating phase. In the last step of the process, compression is applied while the first r bits of the state are used as the data output block.

In this investigation, we zero in on the proposed KECCAK type, which is as follows: A cryptographic hash function may be referred to by the notation Keccak-f[1600], where c = 1024, r = 576, and f = 1600 represent the permutation bit width. The state of Keccak-f[1600] is laid out as an array of 5 × 5 channels, where w corresponds to a data length of 64 bits. KECCAK has a round number of 24, and each round executes the five operations denoted by iota (ι), chi (χ), pi (π), rho (ρ) and theta (θ). Simple logical operations and the permutation of bits provide the basis for these procedures.

### 2.2. Fault Attacks

Fault attacks are a powerful tool for cracking unprotected implementations of KECCAK in hardware. The basic premise of this attack is to corrupt the KECCAK process by inserting one or more bit errors or byte faults during execution, and then exploit the corrupted integrity output to deduce the secret message contained in the cryptographic component.

As described in our previous research [10], we evaluated the robustness of the unprotected KECCAK hardware implementation by simulating a series of fault injection attacks.

Experimental results expose that fault attacks are effective against unprotected KECCAK implementations, and that the KECCAK hardware implementation must be secured against them in order to prevent extracting the KECCAK secret message after a certain number of faults have been injected.

## 3. KECCAK Implementation

**Figure 1:**
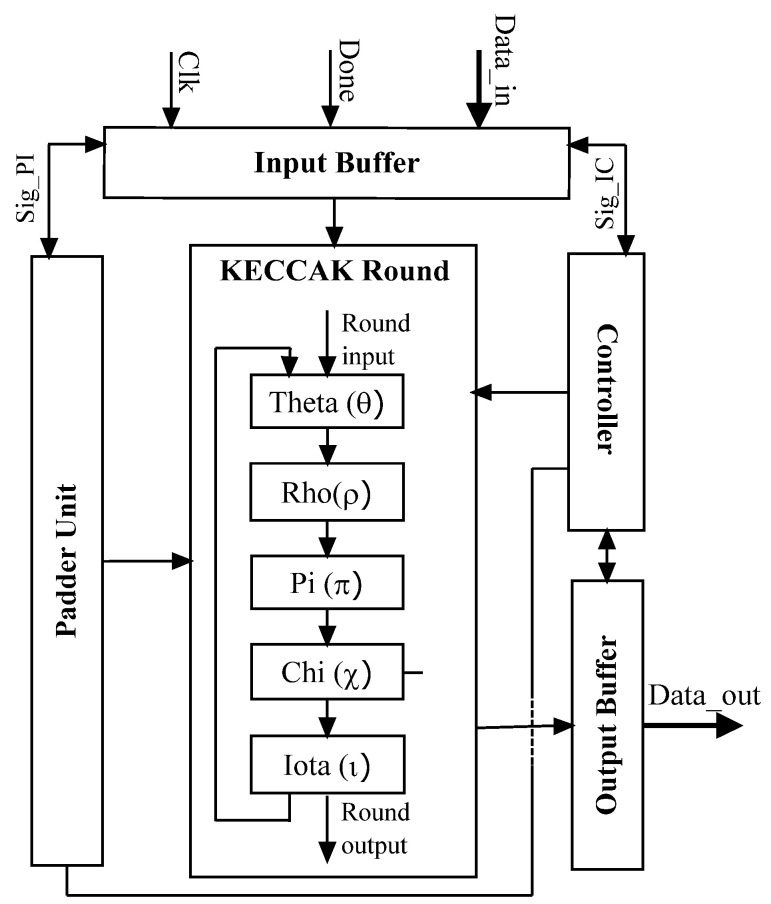
KECCAK architecture.

Figure 1 illustrates the suggested pipelined hardware design for the KECCAK algorithm. The Input Buffer, Padder Unit, Controller, KECCAK Round, and Output Buffer are the five components that make up this design.

- Input Buffer: it ensures KECCAK round-input external module connectivity.
- Padder Unit: executes inversions per byte and performs padding operations. The KECCAK sponge function, which is produced by this module, is 1600 bits in length. A 2-to-1 multiplexer controls the information flow between the Padder Unit and the KECCAK core components.
- The Controller is built to guarantee that all KECCAK modules are synchronized with one another regarding their data transmission.
- KECCAK Round is the core component of KECCAK and runs the 512-bit message digests in 24 clock cycles. The hash output for the current KECCAK Round is calculated using the result from the previous round and the KECCAK constant value.
- Output Buffer: it ensures KECCAK round-output external module connectivity.

Figure 1 shows the KECCAK round’s five operations. Theta, Rho, Pi, Chi, and Iota.

- Theta θ operation: This XORs five columns of input round bits. All state columns are left rotated one bit and XORed again with the preceding operation results. Then, the component’s input lanes are XORed with those results.
- Rho ρ operation: This part carries out a left rotation for each lane’s unique number of positions. The rotation number is found by dividing the component lanes length by the remainder of the fixed value division.
- Pi π operation: This feature was built to adjust the lanes’ location in the Keccak columns as required by the design. In addition, for each row, the component performs logical AND, XOR, and NOT operations on the lanes.
- Chi χ operation: the system is comprised of a matrix consisting of five rows and five lanes, which incorporates a total of 25 XOR, 25 AND, and 25 NOT logic gates, each with a bit capacity of 64.
- Iota ι operation: this component executes an XOR operation on the initial lane (1599-1536) and the round constant value.

We provide KECCAK’s implementation on Xilinx’s Virtex-5 FPGA XC5VFX70T to assess hardware implementation costs. Our KECCAK architecture was modeled in VHDL, and simulated and synthesized with ModelSim 10.1 and Xilinx ISE 14.1, respectively.

The following synthesis results of the proposed KECCAK architecture for FPGA implementation are reported in Table 1:

- Area;
- Frequency;

(1) $$Throughput=\frac{\#bit\times frequency}{\#clockcycles}$$

(2) $$Efficiency=\frac{Throughput}{Area}$$

In addition, Table 1 compares the proposed architecture with three similar reported works [13,14,15,16].

The KECCAK implementation takes 1370 slices for 258.6 MHz frequency. This proposed design achieves 10.77 Gbps throughput for 7.96 Mbps/Slice efficiency. The results indicate that our KECCAK process incurs a reduced area by one fourth and an increased working frequency by 2.3 in comparison to the design proposed in reference [13]. Table 1 presents a comparison between the architecture proposed in this study and the proposed architecture in [14]. Although our architecture has a working frequency less than [14], it allows a higher throughput (10.77 vs. 7.83) and efficiency (7.96 vs. 5.73). Table 1 presents another comparison with the architecture in [15]. This architecture decreases the area and frequency, as well as it reduces the throughput by one half and the efficiency by 1.77 compared to our KECCAK architecture.

## 4. Fault Analysis

In this part, we analyze how fault attacks affect the KECCAK operation. The five round’s procedures performed in the sequence as shown in Figure 1.

Experiments were carried out by putting multiple and single faults into the KECCAK input processes and then counting the number of erroneous bits produced by that operation at its output. These faults might be single or numerous in nature. Because KECCAK processes are carried out a total of 24 times, faults were introduced at random during each round, and the total number of bits that were flawed was determined.

**Figure 2:**
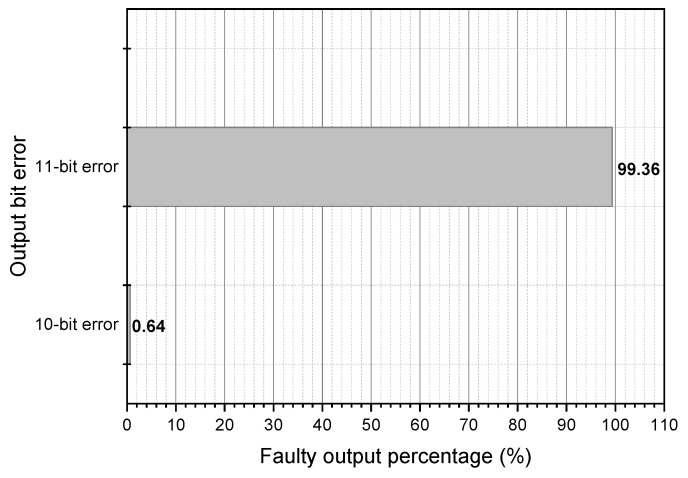
θ operation fault distribution: 1-bit faulty input.

The θ operation is a combination of the XOR and rotate functions. The rotate function modifies the placements of the incorrect bits in the θ output. Therefore, single and multiple faults introduced into the input process result in more than faulty bits in the θ output. Figure 2 illustrates the impact of a single fault attack on the Theta function. The fault is inserted into the input of a single-bit operation. In 99.36% of single fault attack situations, 11 bits of the θ output were flawed.

Two of the 64 operation inputs were defective in the event of a multiple-bit failure attack. The impact of a multiple fault attack on the θ function is presented in Table 2.

The output contains between two and twenty-two defective bits. In 96.67% of fault attack scenarios, 22-bit errors are produced. Error masking occurs in 0% of situations, as indicated by the absence of the zero-error output scenario.

**Figure 3:**
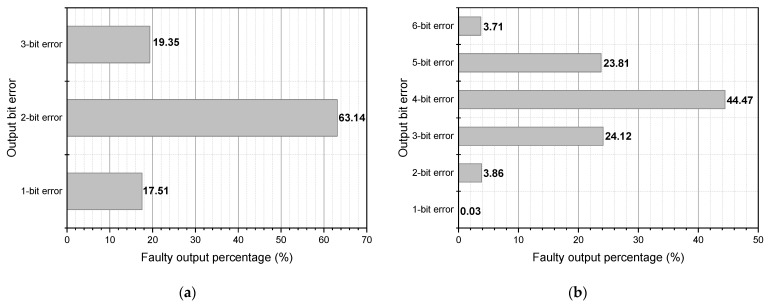
χ operation fault distribution: (**a**) 1-bit faulty input; (**b**) 2-bit faulty input.

The χ operation depends on logic operations (XOR, AND, and NOT) conducted between data processes. Two tests including multiple-bit and single-bit fault attacks were conducted to determine the impact of fault attacks on this procedure. In the first experiment, 1 bit of the χ inputs was affected. Figure 3a depicts the effect of a single-bit fault attack on the χ process. In 63.14% of fault attack scenarios, two-bit errors are produced. The second experiment involved introducing two-bit defects into the χ inputs. As observed in Figure 3b, the number of incorrect bits in the output ranged from 1 bit to 6 bits, indicating that fault masking did not occur in the χ function. In 3.86% and 23.81% of fault attack scenarios, the resulting bit errors are 2 and 5, respectively.

## 5. Fault Detection Scheme

In this part, we outline the motives for this study and then offer a robust fault detection approach for the cryptographic KECCAK algorithm based on modified temporal redundancy. The fault detection technique in this research can be adapted to various of KECCAK.

### 5.1. Motivations

The KECCAK algorithm is the primary one used for information integrity security. This technique may be developed to guard against fault attacks and maintain data integrity. The existence of a flaw detection technique for such a significant cryptographic algorithm may be attributed to two factors.

- Cryptographic circuits are vulnerable to intentional attacks and natural defects, notably, fault injection-based approaches; the KECCAK algorithm is no exception.
- The KECCAK implementation, based on fundamental temporal and hardware redundancy, does the regular hash and re-hash using the same round input, using two clock cycles for each round. In the first cycle, the standard hash is computed, while in the second, the input is re-hashed and the round outputs are compared. In addition, the Tetha θ and Chi χ operations are executed using a protection-based scrambling method. This strategy is efficient since it causes, in case of fault attack, an erroneous integrity message cannot be used to extract the secret message.

### 5.2. Fault Detection Scheme Architecture

**Figure 4:**
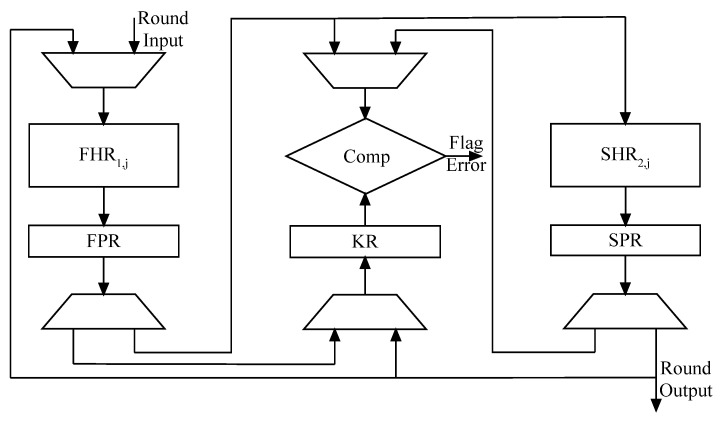
Fault detection scheme architecture.

As seen in Figure 4, the suggested countermeasure is implemented in the KECCAK architecture.

The KECCAK round (KECCAK-512) is executed twenty-four times in order to execute the hash message. Between these two sections, the first pipeline register (FPR) is introduced. Three registers are depicted in Figure 4.

The first pipeline register (FPR) and second pipeline register (SPR) store the intermediate and round’s output values, while the KECCAK register (KR) compares the state messages. The first KECCAK half round (FHR_1,j_) computes the input data’s hash message and saves it in the FPR. The second section (SHR_2,j_) creates outputs that are rounded based on intermediate data. The FHR_1,j_ and SHR_2,j_ critical path delays must the same. Every clock cycle, the stages (FPR, SPR and KR) are run to conduct the KECCAK round and identify any errors. The way registers in Figure 4 are loaded in each clock cycle to perform the round operation and the fault detection process is depicted in Table 3.

The input message is padded and loaded to FKH_1,1_ during the first clock cycle. In the second clock cycle, the first message output of the padded operation is applied to FKH_1,j_. In the third cycle, while SKH_2,j_ executes the result of first KECCAK half round, FKH_1,j_ repeats the FKH_1,1_ hash operation with the same input.

The FKH_1,2_ implementation starts on the fourth cycle, when the SKH_2,1_ is re-executed. The KECCAK register (KR) is used to store the round output and intermediate value for comparison with FPR and SPR stage values, respectively.

Table 3 indicates that:

- The KR register is loaded in even and odd clock cycles to store the output values for comparison.
- The FPR and the SPR registers are loaded in all clock cycles. The stored integrity data in FPR and the SPR are used in even and odd clock cycles, respectively, for error checking.
- The errors checking of the FHR_1,j_ is performed in odd clock cycles.
- The errors checking of the SHR_2,j_ is performed in even clock cycles.

As seen in Figure 4, the FKH_1,j_ (SKH_2,j_ correspondingly) switch alternates between the hash and re-hash processes throughout each clock cycle. At the third cycle of the re-hash technique, the first error checking will be conducted when FKH_1,j_ is compared to the output of the first KECCAK half round. To perform all 24 rounds of KECCAK-512, this method requires 48 clock cycles.

While the hash technique is performed during the second clock cycle, the hash message is not used until the third clock cycle, when the output of FKH_1,j_ is available for fault verification.

The KECCAK hardware architecture involves the duplication of the hashing process data. Hence, two KECCAK rounds execute concurrently. Using the hardware duplication technique, it is straightforward to scramble KECCAK slices between two KECCAK rounds.

We utilized the scrambling method at the conclusion of each KECCAK procedure. In other words, this strategy was utilized at the conclusion of Theta (θ) and Chi (χ). Then, if a problem is introduced into one data hash path, the other data hash path will process data incorrectly.

This solution eliminates fault injection attacks and does not affect the FKH_1,j_ and SKH_2,j_ processes in the absence of attacks, which is an advantage of the proposed architecture. So, we scrambled each bit of the first data hash path with its corresponding bit in the second data hash path in order to strengthen the robustness against fault attacks.

**Figure 5:**
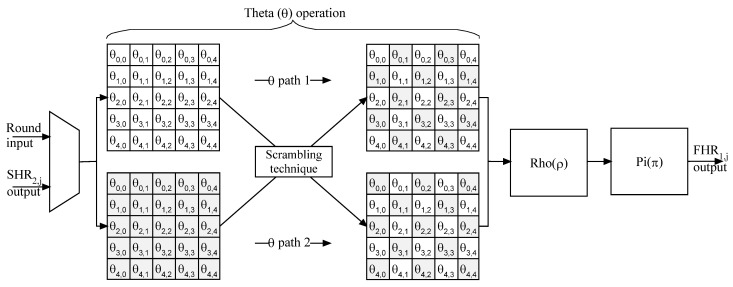
FKH_1,j_ architecture.

**Figure 6:**
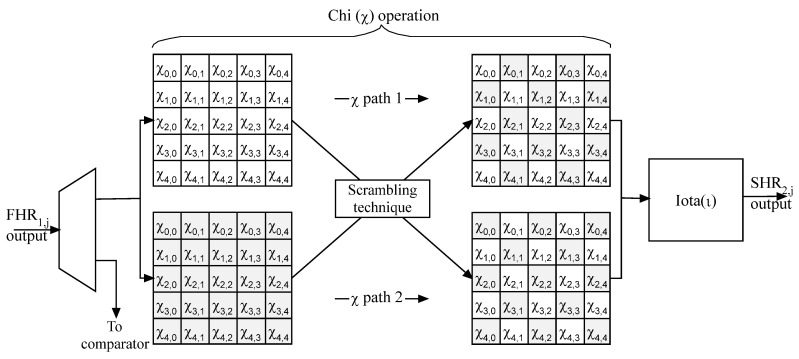
SKH_2,j_ architecture.

As seen in Figure 5 and Figure 6, the architecture details of FKH_1,j_ and SKH_2,j_ are presented.

The slice KECCAK half in data path 1 is mixed up with its corresponding in data path 2. The approach of bit-level scrambling results in a robust KECCAK architecture. In addition, this technique is simple to apply in terms of hardware implementation. Additionally, it does not increase the implementation complexity.

## 6. Fault Detection Evaluation

**Figure 7:**
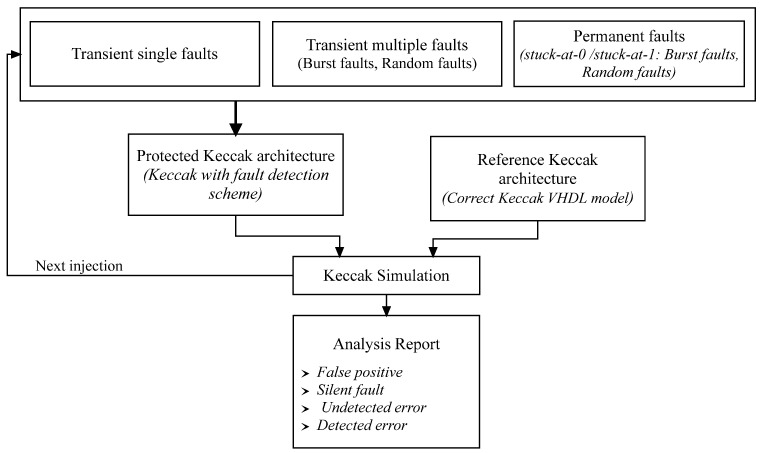
Fault injection/detection process.

To validate the resilience of the proposed design against fault attacks, VHDL simulations were run for our KECCAK fault detection technique. Figure 7 shows the detailed functional description of the fault injection process for evaluating the robustness of the protected KECCAK architecture against three injection fault tests.

The reference cryptographic model is written using VHDL; it presents the correct functional of KECCAK model without injecting faults. The outputs of both modules are passed to the KECCAK simulation that checks them. Then, an analysis report which contains information about the effects of the faults on the proposed designs is generated.

Three injection fault tests are considered:

- Single-bit transient faults: these arise when a single bit of the integrity message is altered.
- Multiple-bit transient faults: these occur whenever there is a change of at least two bits in the integrity state.
- Permanent fault: these alter the hardware design of the KECCAK and can only be fixed by following certain procedures.

Errors are introduced at several target places:

- Injection of errors into the initial messages;
- Injection of errors into all KECCAK rounds FKH_1,j_ and SKH_2,j_;
- Injection of errors into the fault detection data flow, including the error detection flags and comparator;
- Injection of errors into all multiplexers, demultiplexers and registers.

It is noted that the same error can be injected twice in the same position during a single KECCAK round. In addition, we have taken into consideration any and all transient and permanent faults in our architecture. The proposed scheme uses only one error detection flag for KECCAK error verification.

We are able to divide the proposed design’s output into four classes:

- False positive: the round output is the anticipated integrity message, but an inserted error was identified.
- Silent fault: the inserted faults have no effect on our design because the round output is the expected integrity message and no mistake is recognized in the integrity process.
- Undetected error: the detection mechanism failed to identify the occurrence or introduction of faults, despite the fact that the round output was incorrect.
- Detected error: an error is discovered and the output message is different from the expected integrity process, indicating that the fault detection mechanism has identified the occurrence or injection of an error.

In order to lessen the appearance of errors, an effective KECCAK fault detection mechanism is required. It also cannot permit the formation of false positives if the output message is the projected value. The quiet deception is mostly determined by the characteristics of the design.

**Transient single faults:** The suggested fault detection technique was initially tested for its resistance to single-fault assaults. For this fault model, we suppose a single-bit transient fault is injected into one of the aforementioned locations. A total of 2,000,000 errors are used to ensure the security of the simulation. Our simulation findings for the suggested KECCAK architecture’s security are displayed in Table 4.

The proportion of faults discovered is calculated by dividing the total number of single-bit defects injected into the KECCAK architecture by the number of faults detected. Table 4 demonstrates that the vast majority of single-bit transient faults were detected as other types of errors or as false positive. Silence fault only accounted for a minor fraction of all errors (2.354%). Against single-bit transient faults, the rate of undetected errors reaches 0%. This means that our system provides significant protection for KECCAK against fault injection attacks.

**Transient multiple faults:** Multiple-bit transient faults are the primary error model for fault injection assaults, making their fault detection capability extremely significant. The present simulation takes into account two distinct categories of transient faults that affect multiple bits, namely burst faults and random faults.

- Burst faults: This experiment evaluated our KECCAK fault detection technique for its ability to detect errors that impact at least two bits of the integrity message. We introduce transient faults of varying lengths (2-bit–6-bit) into any KECCAK state by injecting groups of erroneous bits. The blunders are introduced into certain specified spots. Consequently, the fault coverage is produced by employing one error detection flag shown in Figure 4. We have used a 2,000,000-multiple-bit evaluation for the suggested KECCAK fault detection technique. Table 4 shows that when a fault is injected at a potential site, it is either false positive (the KECCAK algorithm was implemented and the comparators were subjected to a security breach) or silent fault (rounds 1 through 24 of the KECCAK execution were activated, and the 1st round was affected). With a multiplicity of 6, only roughly 0.0004% of intentionally introduced faults were undetected. When the multiplicity was 2, the percentage of defects that went undiscovered was 0.023%. This indicates that the detection capability increases proportionally with the number of faults.
- Random faults: We inserted 2,000,000 faults with a random fault bit number into the aforementioned locations and observed the results. Table 4 shows that while simulating random-bit transient faults using the suggested KECCAK fault detection technique, 99.9099% of the faults were recognized and the undetected error rate was 0.0001%. If an attacker inserts the same error into two different KECCAK states while adhering to the same restrictions, i.e., the faults are inserted into comparable positions at the same clock cycle, then the undetected fault situation will occur.

**Permanent faults:** In this experiment, we examined the stuck-at-0 and stuck-at-1 faults, wherein the injected faults endure for a duration exceeding a single clock cycle. A total of 2,000,000 faults were injected into all feasible locations. According to Table 4, the undetected error percentage for single-bit permanent faults reached 0%. The preponderance of random permanent faults was categorized as detected errors. Only a tiny fraction of errors was either undetected (approximately 0.000095%) or silent (0.0127%). The results of our fault attacks demonstrate that our design provides a very high degree of protection against attacks that cause permanent faults.

## 7. Hardware Implementation

We provide KECCAK’s implementation on Xilinx’s Virtex-5 FPGA XC5VFX70T to assess hardware implementation costs. Two KECCAK architectures, both unprotected and protected, have been implemented. These KECCAK architectures were modeled in VHDL, simulated and synthesized with ModelSim 10.1 and Xilinx ISE 14.1, respectively.

Table 5 and Table 6 contain the experimental results for the proposed protected and unprotected KECCAK architectures: area and area overhead, frequency and frequency overhead, throughput and throughput degradation, and efficiency and efficiency degradation.

According to Table 5, unprotected KECCAK requires 1370 slices at 258.6 MHz frequency. The protected KECCAK utilizes 22.63% more slices and increases the frequency by 49.65% compared to the unprotected KECCAK design. Due to the divided critical path of the proposed KECCAK architecture, the effective frequency is increased. As shown in Table 5, the working frequency increases by 49.65% compared to unprotected KECCAK. Since the proposed KECCAK design’s critical path is not partitioned into two identical sections, additionally, multiplexers have been incorporated into the KECCAK data path. The protected design has a frequency overhead that is less than twice that of the unprotected architecture.

Compared to the unprotected design, the protected KECCAK degrades throughput by approximately 25.14% and efficiency by approximately 38.26%. Increasing the number of cycles is the primary reason for throughput degradation. The number of unprotected KECCAK clock cycles is 24, while the protected KECCAK requires 48 clock cycles to generate the integrity message.

Table 6 presents a comparative analysis of the proposed architecture with three similar works as reported in references [10,11,12]. The comparison is based on various parameters such as fault coverage (FC), efficiency, throughput, frequency and area overheads. Notably, since most comparable works designate fault outputs as detected errors and undetected errors, we categorized false positives and silent faults as detected errors.

Table 6 presents a comparison between the architecture proposed in this study and the fault detection implementation proposed in [11]. The results indicate that the protected KECCAK process incurs an area overhead of 22.63% and a working frequency overhead of 49.65%. In contrast, the integrity implementation discussed in reference [11] results in a 66.66% area overhead and a 1.75% frequency degradation when compared to the original KECCAK process. The aforementioned statement indicates that our architectural approach incurs a reduced area overhead by one third and an increased working frequency overhead by 51.40% in comparison to the design proposed in reference [11]. Table 6 also demonstrates that our secured KECCAK has a higher FC than the fault detection in [11] at a lower cost, indicating that our fault detection scheme permits a high level of security with comparable throughput and efficiency degradation.

Table 6 presents another comparison with the scheme in [10]. This detection scheme has a slightly lower area overhead and less frequency overhead than our KECCAK. From a security standpoint, the comparison with [10] demonstrates that our proposed KECCAK achieves a higher FC, primarily in random faults (99.9999% vs. 99.997%).

Table 6 indicates that the cost overheads associated with our design are significantly lower than those of [12]. The KECCAK implementation we have presented results in an area overhead of approximately 22.63%. In contrast, the approach described in [12] incurs a significantly higher area overhead of 34.40% compared to unprotected KECCAK. Specifically, the latter KECCAK scheme exhibits an area overhead three times greater than our proposed scheme. Furthermore, our architecture has achieved significantly superior FC in comparison to [12], particularly in the context of random faults (99.9999% vs. 89.89%).

Alvarado et al. [16] proposed a new error detection and correction approach named re-computing for KECCAK. Table 6 compares our architecture with all versions proposed in [16]. The detection schemes in [16] allow frequency and throughput degradation ranges from 31.69% to 45.74% and 63.92% to 69.28%, respectively, which means those schemes allow a frequency degradation up to 2 times and a throughput degradation about 2.75 times those of our architecture. The hardware performances degradation in [16], especially in the area overhead, can be explained by the implementation of both detection and correction schemes.

## 8. Conclusions

In this research, we provided a fault detection technique based on an efficient architectural modification and scrambling technique for the KECCAK algorithm. Our fault simulation faults showed that our detection strategy can detect 99.9999% of transient faults and 99.999905% of permanent faults. In addition, the presented fault detection technique and its counterparts were both implemented using Xilinx Virtex FPGAs. In terms of the KECCAK performance, a comparison was made between their area overhead, frequency overhead, throughput deterioration, and efficiency degradation. The results of the FPGA implementation show that the proposed system may effectively protect the KECCAK implementation against permanent and transient fault attacks, and that it can be easily implemented despite its relatively low complexity. In addition, the experimental results indicate that the frequency overhead is around 44.35%, which is a greater percentage in comparison to previous works that have the same fault coverage. According to the results of our experiments, our suggested method has the maximum efficiency, exhibiting tolerable throughput deterioration as well as area and frequency overheads. This is the case even when the fault coverage is satisfactory. It was shown that the suggested system is more efficient than other previous works in terms of fault detection as well as the hardware implementation cost.

## Figures and Tables

**Table 1:** KECCAK architecture without fault detection scheme: FPGA hardware implementation.

Design	Area (Slice)	Freq. (MHz)	Throu. (Gbps)	Eff. (Mbps/Slice)
[13]	5363	110	-	-
[14]	1365	326.38	7.83	5.73
[15]	1192	223	5.35	4.49
Proposed	1370	258.6	10.77	7.96

**Table 2:** θ operation fault distribution: 2-bit faulty input.

**Output Bit Error Number**	2	11	12	19	20	21	22
**Faulty output (%)**	0.169	0.229	0.642	0.02	1.05	1.22	96.67

**Table 3:** Proposed architecture execution process.

Clock Cycle	Registers Operations	FHR_1,j_	SHR_2,j_
1	Data loading		
Even cycles	KR←FPR Flag Error ← KR ⊕ SPR	Hash	Re-Hash
Odd cycles	KR ← SPR Flag Error ← KR ⊕ FPR	Re-Hash	Hash

**Table 4:** KECCAK architecture: fault evaluation.

Type of Faults				Fault Coverage (%)			
				Silent Fault	False Positive	Undetected Error	Detected Error
Transient faults	Single-bit (N = 1)			2.354	6.569	0	91.077
	Multiple-bit	Burst faults	N = 2	1.047	3.632	0.023	95.298
			N = 3	0.547	1.548	0.014	97.891
			N = 4	0.205	0.759	0.0027	99.0333
			N = 5	0.956	0.395	0.0010	98.648
			N = 6	0.01	0.124	0.0004	99.8656
		Random faults		0.015	0.075	0.0001	99.9099
Permanent faults			Single-bit	1.612	5.142	0	93.246
			Random faults	0.0127	0.059	0.000095	99.928205

**Table 5:** KECCAK architecture: FPGA hardware implementation.

Design	Area (Slice) *(Overhead)*	Freq. (Mhz) *(Overhead)*	Throu. (Gbps) *(Degradation)*	Eff. (Mbps/Slice) *(Degradation)*
KECCAK unprotected	1370	258.6	10.77	7.96
KECCAK protected	1680 (22.63%)	387 (49.65%)	8.06 (25.14%)	4.91 (38.26%)

**Table 6:** KECCAK fault detection implementation: comparison (decrease is denoted by using ‘-’ sign).

Refs.	FC (%)		Overhead (%)			
	Sing-Bit	Rand-Bit	Area	Frequency	Throughput	Efficiency
[10]	99.458	99.997	18.59	44.69	−26.20	−37.72
[11]	99.09	99.996	66.66	−1.75	−1.77	−38.59
[12]	-	89.89	60	-	-	-
[16] ^a^	-	-	823.78	−45.74	−63.92	-
[16] ^b^	-	-	823.71	−31.69	−69.28	-
Proposed	100 *	99.9999 *	22.63	49.65	−25.14	−38.26

* Equal to: detected error + silent fault + false positive; ^a^ architecture version 1 in [16]; ^b^ architecture version 2 in [16].

## Data Availability

Not applicable.
